# Cocoa Pod Husk Pectin Intended as a Pharmaceutical Excipient Has No Adverse Effects on Haematological Parameters in Sprague Dawley Rats

**DOI:** 10.1155/2018/1459849

**Published:** 2018-05-31

**Authors:** Ofosua Adi-Dako, Kwabena Ofori-Kwakye, Kennedy Kwami Edem Kukuia, Jerry Asiedu-Larbi, Alexander Kwadwo Nyarko, Doris Kumadoh, Grace Frimpong

**Affiliations:** ^1^Department of Pharmaceutics, Faculty of Pharmacy and Pharmaceutical Sciences, College of Health Sciences, Kwame Nkrumah University of Science and Technology (KNUST), Kumasi, Ghana; ^2^Department of Pharmaceutics and Microbiology, University of Ghana School of Pharmacy, Legon, Accra, Ghana; ^3^Department of Pharmacology and Toxicology, University of Ghana School of Pharmacy, Legon, Accra, Ghana; ^4^Centre for Plant Medicines Research, Mampong-Akwapim, Ghana; ^5^Department of Pharmaceutical Sciences, Kumasi Technical University, Kumasi, Ghana

## Abstract

Natural polymer research has recently become the focus of intensive research in the quest for new enabling excipients for novel drugs in pharmaceutical formulation for optimal treatment outcomes. Evaluations of some excipients have shown deleterious haematological effects of varying extents on the safety profile of these excipients. A 90-day subchronic toxicity study was conducted to evaluate the influence of cocoa pod husk (CPH) pectin on indicators for haematotoxicity. Male and female Sprague Dawley rats (SDRs) were fed with CPH pectin in doses up to 71.4 mg/kg. The effects of CPH pectin on the haematological indices, direct and total bilirubin, and the spleen were determined. The results indicated that CPH pectin did not induce any untoward toxic effects on the haematological indices, bilirubin levels, and the spleen. There were, however, elevations in MCV at day 30, which was not sustained after the 90 days. The data obtained from this study did not reveal any remarkable findings of toxicological relevance to the haematopoietic system.

## 1. Introduction

Pharmaceutical excipient development is important in the optimization of pharmaceutical formulation and drug delivery [1]. The quality of medicines depends also on the performance of excipients and not only on the active substances [2]. Excipients tend to play multifunctional roles in modern pharmaceutical dosage forms [3]. A promising direction in the quest for novel excipients is in natural polymer material research. Natural products are economical, readily available, capable of modifications, biocompatible, and biodegradable and have an eco-friendly profile [1, 4].

The development of natural polymers as excipients has attracted much interest, as they have unique properties that can be tailored for diverse applications based on their large chains and functional groups. In addition, they can be modified with low and high molecular weight materials to achieve new materials with varied physicochemical properties [1, 5]. Consequently, the successful development of these polymers as excipients has yielded various applications including matrix controlled system, film coating agents, buccal films, microspheres, nanoparticles, implants, viscosity enhancers, stabilisers, disintegrants, solubilisers, emulsifiers, suspending agents, gelling agents, and binders in drug formulation [6].

Cocoa is an important agricultural and economic crop in Ghana, West Africa. Processing of the cocoa pod husks (CPHs) after removing the cocoa beans to useful products like pectin is a means of adding value to the pod husk waste generated [7, 8]. Pectins are natural polymers containing linear chains of (1, 4)-linked *α*-*d*-galacturonic acid residues, with methyl esters of uronic acid [9]. CPH pectin is extracted from pod husk waste after processing of cocoa beans. A previous study reports CPH pectin as having the requisite physicochemical characteristics to be used as a multifunctional pharmaceutical excipient with remarkable properties [10]. The CPH pectin has a moisture content of 0.19 ± 0.06%, ash value of 1.0%, degree of esterification of 26.8 ± 2.5% (low-methoxyl pectin), and swelling index of 357.3 ± 4.6, 274.7 ± 4.6, and 360.0 ± 0.0 in 0.1 M HCl, phosphate buffer pH 6.8, and distilled water, respectively [10]. A study by Yapo et al. indicated that CPH pectin has an intrinsic viscosity of 162–304 mL/g and an average molecular weight of 43–82 kDa under the specified extraction conditions [11].

Chronic diseases such as those influenced by the circadian rhythm are better managed by controlled release formulations than by conventional formulations, which yield a suboptimal treatment outcome, hence the need for such suitable controlled release formulations for optimal drug delivery and enhancement of patient compliance. Pectin has been investigated as matrix controlled systems [6, 12]. Initial exploratory investigations demonstrated the usefulness of CPH pectin as a rate controlling polymer in controlled release formulations for chronic diseases and chronic conditions influenced by the circadian rhythm.

Some chronic conditions of the liver, kidney, and adrenal insufficiency are associated with comorbidities and reports of adverse haematological effects such as macrocytosis, iron deficiency, poor utilization of red cell iron, depressed formation of red cells, and reduced saturation of iron binding protein [13], anaemia [14], and thrombocytopenia [15]. White blood cell count and haemostatic factors play a role in cardiovascular disease, involving disorders of the heart and blood vessels [16]. Similarly, abnormal granulocytic counts had an influence on chronic airways disease [17]. Furthermore, the use of certain herbal preparations for the treatment of such chronic conditions has resulted in some harmful effects such as haematuria from such preparations [18]. Consequently, the treatment of such chronic conditions should not be associated with synergistic or additive effects and a higher risk of further vulnerability to disease resulting from excipients. The effects of excipients should not also confound the diagnosis of other haematological disorders in this regard.

Drug is administered and absorbed to be transported by the blood to the site of action [19]. It is essential that the function of haematological indices like haemoglobin and disease fighting cellular components (e.g., leucocytes) and the blood coagulation system are not impaired in anyway by the excipients present in the drug formulation.

Haematological evaluation of some excipients has shown them to be associated with erythrocyte destruction and the potential for haemolysis, incidences of leucopenia and lymphocytopenia [20], leukaemia [21], and a reduction in haemoglobin and haematocrit after administration of some solubilizing enhancing agents [22]. In addition, the toxicity profile of the excipient is influenced by the route of administration [22].

In view of these, it is essential that the enabling excipients for the drug formulation in the treatment of such chronic conditions should have the desired safety profile for continuous long-term administration. In the past, excipients were considered as components of a pharmaceutical dosage form, aside of the active ingredient, which were “inert.” This concept of excipients has evolved over time, as they are now regarded as functional components of pharmaceuticals [2, 23]. Excipients could be of animal, plant, or mineral origin. The variation in the sources of natural excipients requires adequate quality assurance and safety evaluation to be undertaken. Previous reports indicate biological activities of pectin to include ability to reduce blood cholesterol levels, ability to combat heavy metal poisoning of the blood, and having haemostatic and antidiarrhoeal action [24, 25].

Moreover, excipients are not always inert with regard to their biological behavior. Excipients of natural origin are capable of exhibiting biological activity [26] which could invariably affect their safety profile [21]. Another concern is related to the extraction of such natural polymers, which could be accompanied with other trace phytochemical and biological constituents [23].

The limited information available on the effect of CPH pectin on the haematological profile necessitates an evaluation after long-term oral continuous administration on the major indices of haematological toxicity in Sprague Dawley rats (SDRs) [27]. Thus, the study investigated the effect of CPH pectin on some haematological indices, bilirubin, and also the histology of the spleen.

## 2. Materials And Methods

### 2.1. Materials

Freeze-dried CPH pectin with degree of esterification of 26.8% extracted with hot water (hot water soluble pectin, HWSP) and 4% w/v hot aqueous citric acid (citric acid soluble pectin, CASP), as described elsewhere [10], were used for the study.

### 2.2. Animal Husbandry and Maintenance

Healthy male and female Sprague Dawley rats, approximately 6 weeks old, were obtained from the Animal House of the Centre for Plant Medicine Research (CPMR), Mampong-Akwapim, in the eastern region of Ghana. The animals were housed in stainless steel wire mesh cages with soft wood shavings under experimental conditions (temperature 22 ± 2°C, relative humidity 60–70%, and 12 h light-dark cycle) and fed with feed from Agricare Co., Ltd., Accra, Ghana. The animals had access to clean water* ad libitum*. The study was conducted in accordance with the Institutional Research Committee of the CPMR responsible for Animal Care and Use, as well as the National Institute of Health Guidelines for the Care and Use of Laboratory Animals as found in the US guidelines [28].

### 2.3. Animal Grouping And Treatment

Twenty-four male Sprague Dawley rats were randomly grouped into four experimental groups (*n* = 6) according to weights. The control (Group A) was given distilled water orally at 5 ml daily for the duration of 90 days, the second set of test animals (Group B) was given 0.714 mg/kg of CPH pectin, the third set (Group C) was given 7.14 mg/kg of CPH pectin, and the last set (Group D) was given 71.4 mg/kg of CPH pectin, all by oral gavage. The experiments were repeated with twenty-four female rats placed in four groups (*n* = 6). It was ensured that the female rats were nulliparous and not pregnant. All the animals received treatment for the 90-day period. The animals were observed and monitored daily for mortality and any clinical signs of toxicity or abnormality.

### 2.4. Body Weights

Each rat in the experimental groups was weighed on day 0 and then weekly for the 90-day study period for each of the dose levels.

### 2.5. Food And Water Consumption

The food and water consumed by each rat were monitored for the 90-day period.

### 2.6. Haematology

Blood samples were taken from each rat via tail bleeding into Eppendorf tubes containing EDTA for haematological analysis the same day. The RBC, WBC, HGB, HCT, MCV, MCH, MCHC, RDW-SD, and RDW-CV% were measured with an automated haematology analyzer (KX-2IN, Sysmex Corporation, Japan). This was done every 30 days over the 90-day period.

### 2.7. Serum Biochemistry

The animals in each group were fasted overnight before blood collection the next day. Blood samples for the biochemical analysis were collected into BD-serum separator tubes. The blood samples were then centrifuged at 3000 rpm for 10 minutes. The serum obtained was then stored at 4°C and used later for analysis of direct and total bilirubin the next day. The analysis was conducted every 30 days over the 90-day period.

### 2.8. Animal Sacrificing

A trained expert euthanized the rats by cervical dislocation on the same day the experiment was performed.

### 2.9. Histopathology

Tissue obtained from the spleen was preserved in 10% formalin. The spleen tissue was then processed, embedded in paraffin, and sectioned at 3–5 um. After this, the sections were stained with haematoxylin-eosin for the microscopic examination [29].

### 2.10. Statistical Analysis

Statistical analysis of the data was done using GraphPad Prism version 5.03. The results obtained were expressed as mean ± standard error of mean (SEM) (*n* = 6). Significant differences between dose groups and controls were evaluated by performing a two-way analysis of variance (ANOVA) and a one-way ANOVA. If the test showed significant differences, the Bonferroni or Dunnett's* post hoc* analysis was performed, respectively.* P* value less than 0.05 was considered significant.

## 3. Results

### 3.1. Mortality

There was no mortality associated with the study.

### 3.2. Food and Water Consumption

There was no significant variation in the food and water consumption among the groups under study.

### 3.3. Effect of CPH Pectin on Weight Change in Male and Female Sprague Dawley Rats

There was a consistent weight gain (P>0.05) in male rats. There were, however, no dose-specific patterns in body weight related to treatment. Female rats demonstrated rapid weight gain during the first four weeks and a slower rate thereafter. There were slight changes in female body weight (*P* < 0.05) at week 13 prior to termination. There were no dose-specific patterns in body weight related to treatment (Table 1).

### 3.4. Effect of CPH Pectin on RBC Indices of Male and Female Sprague Dawley Rats

Table 2 shows the influence of the administration of CPH pectin on haematological indices for the male rats after a 90-day subchronic toxicity study. In comparison to the control, treated animals showed no remarkable changes with *P* > 0.05 except for the MCV value for male rats treated with medium dose (7.14 mg/kg) after day 30 (*P* < 0.01). All other parameters showed similar results at 30 and 60 days except for MCV. Values for MCV indicated an isolated statistically significant reduction with the medium dose group after 30 days.  Table 3 shows the influence of the administration of CPH pectin on haematological indices for the female rats after a 90-day subchronic toxicity study. In comparison to the control, treated animals showed no remarkable changes. All other parameters showed similar results at 30 and 60 days except for MCV.

### 3.5. Effect of CPH Pectin on WBC Indices of Male and Female Sprague Dawley Rats

Table 4 shows the effect of CPH pectin on the levels of leucocytes, lymphocytes, and neutrophils in male SDRs for the 90-day subchronic toxicity study. Minor fluctuations were observed in both control and treated groups during the 90-day administration of CPH pectin with no marked effects (*P* > 0.05) on these white blood cell indices. Similarly, there were minor fluctuations observed for the female rats in both control and treated groups during the 90-day administration of CPH pectin (Table 5).

### 3.6. Effect of CPH Pectin on Platelet Indices of Male and Female Sprague Dawley Rats

Analysis of platelet indices (Table 6) showed no significant changes (*P* > 0.05) in platelet count (PLT), though there were insignificant minor reductions after day 30 in male rats. Similarly, there were no remarkable changes in the ratio (P-LCR) and size (PDW and MPV) after the 90-day continuous administration of CPH pectin. Again, analysis of platelet indices in female rats (Table 7) showed no significant changes (*P* > 0.05) in platelet count (PLT). There was minor reduction of no significance after day 30 in the female rats. Similarly, there were no remarkable changes in the ratio (P-LCR) and size (PDW and MPV) after the 90-day continuous administration of CPH pectin

### 3.7. Effect of CPH Pectin on Bilirubin Levels of Male and Female Sprague Dawley Rats

Table 8 shows the influence of CPH pectin on direct and indirect bilirubin levels after a 90-day continuous administration of CPH pectin in male SDRs. In comparison to the control, there were insignificant elevations in bilirubin at day 30 (*P* > 0.05) in the treated groups. There were no remarkable changes observed in bilirubin levels for days 60 and 90 (*P* > 0.05). The effect of CPH pectin on indirect bilirubin levels showed insignificant elevations in bilirubin at day 30 (*P* > 0.05) in the treated groups. There were no remarkable changes observed in the levels for days 60 and 90 (*P* > 0.05).

Similarly, in the female rats (Table 9), there were generally insignificant reductions in direct bilirubin levels with no dose-specific pattern. There were no remarkable changes observed in bilirubin levels for the 90-day study period (*P* > 0.05). In comparison to the control, there were no remarkable changes observed in indirect bilirubin levels for the 90-day study period in female rats as well (*P* > 0.05).

Table 10 shows the effect of CPH pectin on total bilirubin levels after a 90-day continuous administration of CPH pectin in male SDRs. In comparison to the control, there were insignificant elevations in total bilirubin at day 30 (*P* > 0.05) in the treated groups. Generally, there was a reduction in total bilirubin levels observed for days 60 and 90 (*P* > 0.05) with no dose-specific pattern. There were no remarkable changes observed in total bilirubin levels. Again there were generally insignificant reductions in total bilirubin levels (P > 0.05) with no dose-specific pattern in female rats

### 3.8. Effects of CPH Pectin on the Spleen of the Female SDR Spleen

**Figure 1:**
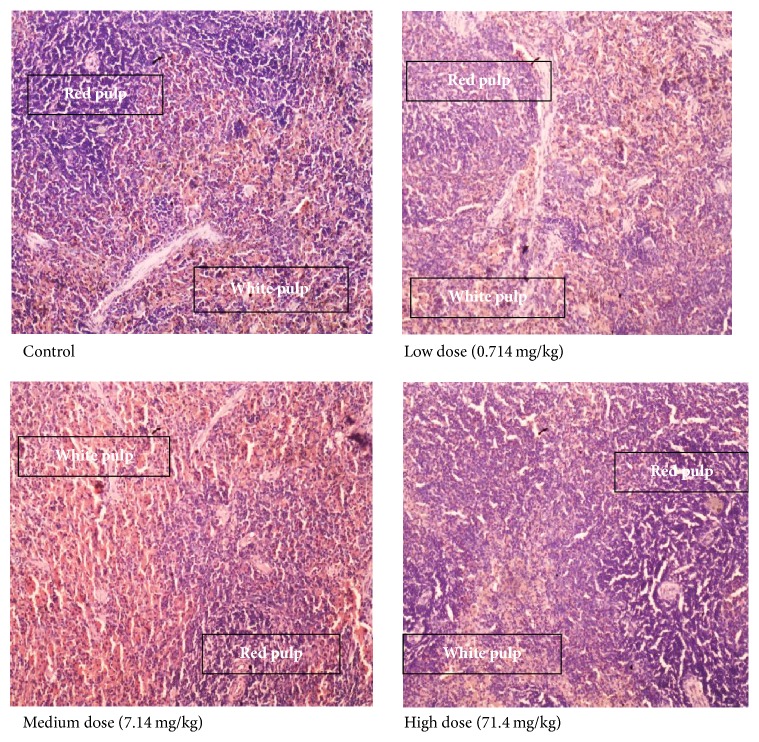
The effect of varying dose levels of CPH pectin on the histology of the spleen of female SDRs (magnification, ×400).

Figure 1 shows tissues from the spleen with normal white pulp, consisting of lymphocytes, and normal red pulp, consisting of vessels with red blood cells. There are regular lymphoid aggregates.

## 4. Discussion

Essential oils, extracted from the seeds of* Simmondsia chinensis,* have been reported to be beneficial in formulation as enhancers in matrix transdermal patches [30], emulgels [31], and microemulsions [32]. However, there seem to be adverse reports associated with suppression of bone marrow with severe anaemia, profound weight loss, and death with a high dose of a constituent of the plant* S. chinensis* in chronic feeding studies in rats [33]. Data from preclinical toxicity studies are highly beneficial for the determination and evaluation of the effects of potential excipients on body weight and haematology for formulation development.

Eisele et al. reported on an associated low weight gain with the administration of a copolymer excipient, due to the swelling behaviour of the test material [34]. CPH pectin had similar swelling capacity and a viscous nature useful for its gelling ability and application in formulation [10] which could create a feeling of satiety. However, the 90-day oral continuous administration of CPH pectin did not influence the male rat body weight.

Female rat weight gain was rapid for all dose levels within the first four weeks and at a slower rate thereafter. There were no toxicologically relevant changes in body weight. This suggests that CPH pectin did not impair carbohydrate or fat metabolism as well [35]. It is also plausible that the presence of certain bioactives from cocoa pod husk has a positive influence on carbohydrate and lipid metabolism. Cocoa polyphenols are present in the cocoa pod husk. As many phenolic compounds are water-soluble, minimal quantities could be extracted together with CPH pectin [10, 36]. Cocoa polyphenols were reported to improve the cholesterol profile of patients. In this study, body weight, inflammatory markers, insulin resistance, and glycaemic control were not affected [37].

Moreover, there was no indication of any abnormal signs of toxicity in the rats under investigation, such as piloerection, cyanosis, paralysis, drowsiness, sedation, or gastrointestinal problems such as diarrhea.

Haematological preclinical toxicity studies are essential to identify test compounds that can exert toxic effects on the cellular constituents of blood (e.g., red blood cells (erythrocytes), white cells (leucocytes), and platelets). Any observed toxic responses could result from the direct effect of the test compound on the circulating cells. Haemolytic anaemias have been reported after subsequent red blood cell destruction or an interference with their production and/or their development. Data from such important studies is able to detect the development of anaemia and gain some insight into the mechanism leading to toxicity [29]. The haematopoietic system consisting of blood cells presents as a peculiar target organ as it is susceptible to damage on exposure to potential toxicants [38].

An evaluation of haematological indices after a 90-day administration of CPH pectin revealed no adverse effects on the red blood cell indices like RBC, HGB, MCH, HCT, and MCHC and was unaffected in both male and female SDRs. However, there was an incidental decrease in the MCV of the male rats after day 30 when rats received the medium dose of the CPH pectin. Balan and Veretiuc reported on the haemolytic activities of some polymer excipients [39], while other investigators reported excipient-induced haemagglutination due to the formation of polyelectrolyte complexes [40, 41]. Fernandes et al. also reported the deleterious effect of the degradation products of polymeric excipients on human erythrocytes resulting in adhesion and aggregation of the blood cells [42]. Similarly, Soffriti et al. previously reported an increase in the incidence of some blood disorders associated with the administration of excipients, useful as sweetening agents, in pharmaceutical formulation, in male Sprague Dawley rats [43]. We considered the observed changes in MCV associated with CPH pectin to be transient or incidental and of no adverse consequence as the observation was absent in the low and high dose groups and over time. In addition, the changes fell in the normal range of historical control data and were therefore of no toxicological significance [44].

The findings showed no evidence of decreased haemoglobin synthesis or increased red cell destruction and no indication of macrocytic or microcytic anaemia [37]. Repeated dose administration of CPH pectin showed no influence on critical indicators of anaemia, like the RBC count, HGB, and HCT, and hence no treatment-related effects were observed.

Bilirubin is usually produced after the breakdown of erythrocytes and other haem-containing proteins. Liver disorders are, however, associated with jaundice. Hepatocellular jaundice occurs when there is a build-up of bilirubin in the plasma as it is not transported, conjugated, or excreted by the damaged liver cells. Subsequently bilirubin builds up in the plasma as the flow is obstructed in the small bile channels or in the main bile duct. Cholestasis, the disruption of this flow, is associated with hepatocellular damage. In severe haemolysis, more bilirubin is produced than the liver can metabolize, resulting in elevated bilirubin levels in the plasma or serum [45]. There were statistically insignificant elevations in indirect and total bilirubin levels associated with the low and high dose groups of the male rats at day 30. However, this observation was not consistent over the 90-day study period and was not of any toxicological relevance. Hence, direct, indirect, and total bilirubin levels remained unaffected by the 90-day administration of CPH pectin. This was indicative of the absence of any deleterious effects of CPH pectin on the erythrocytes for the 90-day study period.

Drug- or excipient-induced white cell toxicity could be assessed by the measurement of the total number of circulating leucocytes (i.e., WBC count together with an estimation of the percentage of each of the different types of leucocytes, e.g., neutrophils) and lymphocytes present in the sample. A toxicant may cause leucopenia or leukocytosis. Such responses may be associated with one or more cell types (e.g., neutrophilia associated with certain inflammatory reactions) [29]. In this study, the administration of CPH pectin did not cause changes in white blood cells in both male and female SDRs. Similarly, the continuous administration of CPH pectin showed no deleterious effects on neutrophils, suggesting that CPH pectin did not inhibit granulopoiesis or neutrophil function. It also did not cause neutropenia. This also suggests that CPH pectin over the period did not cause neutrophil damage [46–48]. It may be inferred then that CPH pectin did not induce leucopenia, lymphocytosis, or lymphocytopenia [37].

Owing to the importance of various leucocytes in fighting infection and their involvement in immune responses, it comes as a relief that CPH pectin did not induce adverse effects on these white blood cells. Moreover, administration of CPH pectin during the 90-day study showed no adverse immunological implications, unlike other excipients or drugs that exhibit immunotoxic effects [49, 50].

Platelets are susceptible to a variety of toxicants. Drug- or excipient-induced thrombocytopenia results from peripheral destruction or bone marrow suppression. Increased peripheral destruction can result from immune mediated mechanisms or from platelets having a shortened survival time. A drug or excipient may not affect platelet number but could impair platelet function. In this case, platelet counts are normal, but animals may exhibit abnormal bleeding tendencies [29].

There have been reports of polymer excipients that have thrombogenic activity subsequent to adhesion and activation of platelets and blood coagulation [51]. In this study, the platelet count was unaffected by the administration of CPH pectin over the 90-day period. This suggests that CPH pectin had no thrombocytopenic effect and did not exhibit any abnormal bleeding tendencies [29]. There were also no abnormal levels in the biomarkers PDW and MPV and P-LCR to suggest thromboembolic disease after the continuous administration of CPH pectin [52]. The findings also suggest the compliance to good manufacturing practice with respect to the efficiency of extraction, suitable processing, and storage of CPH pectin required for pharmaceutical grade excipients, as there was no indication of the presence of harmful contaminants from adulteration, heavy metal, or microbial contaminants which could result in haematotoxicity [23, 53].

CPH pectin, belonging to the class of polysaccharides, was administered orally throughout the study. There have been previous reports indicative of the negligible absorption of pectin in the gastrointestinal tract when administered orally [54, 55]. Pectin is a soluble dietary fibre and is generally regarded as safe (GRAS) by the FDA, with a wide margin of safety [56]. This suggests that there is limited systemic exposure of CPH pectin, which may account for its observed safety profile even after continuous long-term administration.

The spleen is known to play an important role in the clearance of damaged and aging blood cells as well as enhancing the immune system of the host [37]. There were no treatment- or dose-related microscopic pathological changes in the spleen after administration of CPH pectin over the 90-day study period.

These findings, although in their early days, are significant. This is because CPH pectin has demonstrated safety as natural polymer and thus its future approval and incorporation into pharmaceutical formulation demonstrate the immense benefit of natural polymers as excipients as has been mentioned previously.

## 5. Conclusion

The findings suggest that CPH pectin administered up to 71.4 mg/kg for 90 days showed no evidence of toxicity on the major haematological indices in male and female SDRs.

## Figures and Tables

**Table 1:** Results showing the effect of CPH pectin on body weight change in SDRs.

Week	Control	0.714 mg/kg	7.14 mg/kg	71.4 mg/kg
	Male			

0	161.00±4.62	163.00±4.70	161.67±3.39	171.30±4.13
4	209.50±6.46	230.83±6.17	229.33±4.05	227.50±4.80
8	234.17±4.48	260.00±7.90	256.87±8.65	249.50±5.50
13	267.67±16.87	272.50±12.26	266.17±12.36	270.30± 10.00
	Female			

0	133.00±1.24	162.67±0.88	153.17±0.98	117.83±0.79
4	173.70±5.29	179.17±1.49	173.83±2.17	161.50±2.88
8	186.83±6.93	184.50±3.37	182.00±0.45	180.67±4.24
13	200.50±7.84	190.83±1.49	185.50±2.26	189.00±4.40

**Table 2:** Results showing the effect of CPH pectin on red blood cell indices of male rats.

Red blood cell indices	Day	Control	Low dose 0.714 mg/kg	Medium dose 7.14 mg/kg	High dose 71.4 mg/kg
RBC (x 10^6^/ul)	30	8.72±0.09	9.16±0.23	8.93±0.14	8.91±0.13
	60	8.78±0.07	8.68±0.12	8.58±0.18	8.60±0.11
	90	9.03±0.11	8.90±0.12	8.77±0.15	8.91±0.14

HGB (g/dl)	30	15.00±0.18	15.52±0.36	15.63±0.43	15.48±0.16
	60	15.37±0.20	15.18±0.34	15.03±0.22	15.08±0.12
	90	15.50±0.13	15.38±0.34	15.35±0.27	15.35±0.18

HCT (%)	30	49.47±0.59	49.68±0.68	49.87±1.07	48.52±0.61
	60	47.48±0.67	47.10±1.21	45.87±0.83	46.88±0.68
	90	48.82±0.44	49.97±0.97	47.62±0.88	50.30±0.78

MCV (fl)	30	55.60±0.19	54.92±0.62	52.95±0.26*∗∗*	53.73±0.35
	60	53.20±0.31	52.65±0.57	53.50±0.36	54.23±0.40
	90	54.08±0.70	53.50±1.11	54.83±0.53	55.88±0.49

MCH (pg)	30	17.08±0.18	16.93±0.26	16.98±0.22	17.15±0.13
	60	17.32±0.11	17.10±0.26	17.52±0.16	17.55±0.12
	90	17.05±0.11	16.92±0.21	17.47±0.39	17.47±0.11

MCHC (g/dl)	30	30.63±0.25	30.53±0.18	31.90±0.19	31.63±0.12
	60	32.37±0.13	32.23±0.19	32.80±0.16	32.18±0.29
	90	31.83±0.12	31.22±0.59	31.60±0.85	30.90±0.18

RDW-SD (fl)	30	29.55±0.26	29.55±0.30	28.00±0.39	27.75±0.40
	60	27.50±0.24	27.03±0.27	27.52±0.07	27.68±0.31
	90	28.55±0.91	29.87±1.28	28.23±0.76	29.83±0.45

RDW-CV (%)	30	13.55±0.56	14.70±0.77	14.57±0.38	12.53±0.39
	60	13.03±0.63	13.08±0.58	13.12±0.33	13.03±0.36
	90	14.30±1.14	15.33±1.40	13.57±0.57	13.58±0.50

*∗∗*Significantly different (*P* < 0.01)

**Table 3:** Results showing the effect of CPH pectin on red blood cell indices for female rats.

Red blood cell indices	Day	Control	Low dose 0.714 mg/kg	Medium dose 7.14 mg/kg	High dose 71.4 mg/kg
RBC (x 10^6^/ul)	30	8.13±0.10	8.36±0.18	7.72±0.19	8.25±0.11
	60	7.77±0.06	8.15±0.15	7.96±0.14	7.88±0.14
	90	8.08±0.10	8.14±0.21	8.14±0.17	8.26±0.12

HGB (g/dl)	30	15.40±0.12	14.57±0.23	14.20±0.26	14.98±0.08
	60	14.95±0.13	14.87±0.27	14.27±0.29	14.62±0.15
	90	15.18±0.19	14.72±0.28	14.82±0.23	15.18±0.19

HCT (%)	30	43.80±0.47	42.88±0.40	41.42±0.86	43.48±0.40
	60	42.75±0.39	43.95±0.69	41.97±0.60	42.83±0.54
	90	44.30±0.94	43.87±0.87	44.22±0.68	44.97±0.65

MCV (fl)	30	53.85±0.61	52.35±0.35	53.70±0.54	52.73±0.39
	60	54.97±0.26	53.93±0.36	54.23±0.44	54.37±0.50
	90	54.72±0.61	53.97±0.68	54.32±0.40	54.42±0.38

MCH (g/dl)	30	18.95±0.14	17.78±0.15	18.38±0.18	18.15±0.17
	60	19.23±0.10	18.23±0.21	17.96±0.50	18.58±0.21
	90	18.82±0.12	18.10±0.26	18.20±0.16	18.38±0.13

MCHC (g/dl)	30	34.97±0.30	34.48±0.26	34.67±0.18	34.80±0.18
	60	34.55±0.12	34.00±0.14	34.32±0.20	34.12±0.16
	90	34.37±0.20	33.63±0.09	33.82±0.22	33.83±0.07

RDW-SD (fl)	30	26.00±0.18	26.22±0.23	25.98±0.21	25.98±0.13
	60	26.68±0.16	26.72±0.22	26.62±0.23	26.62±0.23
	90	26.58±0.20	26.58±0.12	26.75±0.59	26.16±0.10

RDW-CV (%)	30	11.53±0.25	11.37±0.24	11.50±0.79	10.95±0.15
	60	11.50±0.26	11.70±0.25	12.25±0.92	11.37±0.21
	90	11.35±0.31	11.67±0.09	11.93±0.89	11.17±0.44

**Table 4:** The influence of CPH pectin administration on white blood cell indices for male rats.

White blood cell indices	Day	Control	Low dose 0.714 mg/kg	Medium dose 7.14 mg/kg	High dose 71.4 mg/kg
WBC (x10^3^ ul)	30	14.38±0.56	13.43±0.78	13.03±0.49	13.80±0.59
	60	14.47±1.48	12.60±1.09	13.68±0.80	12.50±1.18
	90	10.63±0.65	10.65±0.67	10.28±0.56	11.13±0.69

LYM (%)	30	76.57±4.80	78.03±3.39	79.28±1.65	75.13±1.38
	60	78.12±2.12	75.20±2.19	78.58±4.23	74.72±7.42
	90	71.95±2.54	67.13±0.63	68.98±4.19	68.60±3.81

NEUT (%)	30	17.33±1.90	17.07±1.05	17.50±1.40	17.52±2.08
	60	15.77±1.58	16.65±1.45	16.45±1.91	14.13±2.88
	90	21.42±2.20	20.00±0.76	19.96±2.80	20.40±2.46

**Table 5:** The influence of CPH pectin administration on white blood cell indices for female rats.

White blood cell indices	Day	Control	Low dose 0.714 mg/kg	Medium dose 7.14 mg/kg	High dose 71.4 mg/kg
WBC (x10^3^ ul)	30	12.47±1.00	13.13±1.98	15.20±1.40	13.73±1.16
	60	14.98±1.05	15.55±2.10	16.72±1.21	14.40±1.20
	90	13.80±1.45	13.85±0.55	15.17±1.52	12.77±0.97

LYM (%)	30	70.57±3.22	67.32±2.63	70.50±4.21	71.58±2.10
	60	68.23±3.17	70.07±2.96	65.85±6.92	74.78±3.29
	90	69.20±3.22	70.37±3.93	66.00±2.84	67.17±2.16

NEUT (%)	30	20.42±1.17	20.47±1.97	21.30 ±2.19	21.78±1.90
	60	20.62±2.53	23.27±2.95	21.93±2.97	18.72± 1.45
	90	22.27±2.58	24.45±3.50	22.33±1.68	22.48±2.12

**Table 6:** The influence of CPH pectin administration on platelet indices for male rats.

Platelet indices	Day	Control	Low dose 0.714 mg/kg	Medium dose 7.14 mg/kg	High dose 71.4 mg/kg
PLT (x10^3^/ul)	30	1019.83±42.53	1033.50±38.47	1013.17±47.30	1046.83±48.54
	60	837.33±23.61	884.83±63.93	924.67±43.98	969.50±41.36
	90	881.83±49.09	923.67±63.82	972.83±54.03	1015.50±45.94

PDW (fl)	30	8.65±0.26	8.83±0.24	8.88±0.11	9.00±0.27
	60	9.12±0.14	9.62±0.36	9.57±0.11	9.47±0.35
	90	9.05±0.27	9.20±0.29	9.25±0.13	9.00±0.25

MPV (fl)	30	7.33±0.13	7.40±0.15	7.40±0.06	7.47±0.16
	60	7.47±0.14	7.68±0.17	7.77±0.10	7.73±0.21
	90	7.40±0.13	7.45±0.12	7.60±0.06	7.55±0.14

P-LCR (%)	30	6.62±1.32	7.78±0.86	7.57±0.30	8.42±0.89
	60	9.10±0.62	10.35±1.03	9.90±0.43	10.02±1.30
	90	8.18±0.74	8.37±0.74	9.28±0.39	8.63±0.94

**Table 7:** The influence of CPH pectin administration on the platelet indices for female rats.

Platelet indices	Day	Control	Low dose 0.714 mg/kg	Medium dose 7.14 mg/kg	High dose 71.4 mg/kg
PLT (x10^3^/ul)	30	1046.30±42.90	1096.70±45.60	1046.50±53.20	1070.00±42.30
	60	981.30±49.90	1033.20±51.00	1081.00±61.00	942.80±32.40
	90	1097.00±73.80	1048.70±55.20	975.50±54.80	959.80±20.20

PDW (fl)	30	8.30±0.12	8.62±0.17	8.57±0.11	8.65±0.09
	60	8.48±0.08	8.57±0.24	8.65±0.23	8.48±0.15
	90	8.37±0.14	8.27±0.03	8.50±0.10	8.33±0.11

MPV (fl)	30	7.00±0.07	7.10±0.05	7.10±0.09	7.18±0.08
	60	7.07±0.07	7.20±0.17	7.12±0.14	7.05±0.06
	90	7.07±0.09	6.98±0.05	7.10±0.10	7.02±0.10

P-LCR (%)	30	6.73±0.30	7.08±0.24	7.18±0.31	7.93±0.55
	60	7.23±0.37	7.25±0.34	7.60±0.57	7.70±0.20
	90	7.37±0.33	5.95±0.19	6.38±0.32	6.53±0.43

**Table 8:** Results of the effects of CPH pectin on direct and indirect bilirubin in male SDRs.

Day	Control	0.714 mg/kg	7.14 mg/kg	71.4 mg/kg
	Direct bilirubin			

30	0.63±0.13	0.80±0.29	0.41±0.13	0.88±0.17
60	0.50±0.17	0.32±0.12	0.38±0.14	0.47±0.36
90	0.77±0.22	0.46±0.10	0.54±0.24	0.72±0.26
	Indirect bilirubin			

30	1.21±0.21	1.64±0.39	1.28±0.20	1.73±0.40
60	0.25±0.19	0.12±0.08	0.26±0.21	0.20±0.72
90	0.20±0.18	0.18±0.20	0.25±0.17	0.11±0.26

**Table 9:** Results of the effects of CPH pectin on direct and indirect bilirubin in female SDRs.

Day	Control	0.714 mg/kg	7.14 mg/kg	71.4 mg/kg
	Direct bilirubin			

30	0.50±0.24	0.45±0.17	0.41±0.06	0.36±0.07
60	0.55±0.21	0.54±0.17	0.40±0.14	0.34±0.11
90	0.73±0.30	0.49±0.26	0.64±0.25	0.60±0.16
	Indirect bilirubin			

30	0.42±0.27	0.35±0.04	0.30±0.06	0.44±0.10
60	0.35±0.15	0.31±0.14	0.29±0.09	0.27±0.06
90	0.39±0.18	0.40±0.21	0.28±0.15	0.25±0.16

**Table 10:** Results showing the effects of CPH pectin on total bilirubin in male and female SDRs.

Day	Control	0.714 mg/kg	7.14 mg/kg	71.4 mg/kg
	Male			

30	1.84±0.19	2.44±0.22	1.68±0.27	2.61±0.25
60	0.75±0.25	0.44±0.16	0.64±0.23	0.70±0.37
90	0.97±0.27	0.64±0.15	0.78±0.18	0.83±0.32
	Female			

30	0.92±0.07	0.81±0.14	0.72±0.04	0.74±0.07
60	0.91±0.10	0.85±0.14	0.68±0.18	0.62±0.14
90	1.12±0.15	0.89±0.06	0.92±0.13	0.85±0.24
